# Size-Resolved Community Structure of Bacteria and Fungi Transported by Dust in the Middle East

**DOI:** 10.3389/fmicb.2021.744117

**Published:** 2021-11-10

**Authors:** Daniella Gat, Naama Reicher, Shai Schechter, Matan Alayof, Mark D. Tarn, Bethany V. Wyld, Ralf Zimmermann, Yinon Rudich

**Affiliations:** ^1^Department of Earth and Planetary Sciences, Weizmann Institute of Science, Rehovot, Israel; ^2^Joint Mass Spectrometry Centre (JMSC), Comprehensive Molecular Analytics (CMA), Helmholtz Zentrum München – German Research Center for Environmental Health (GmbH), Munich, Germany; ^3^Institute for Climate and Atmospheric Science, School of Earth and Environment, University of Leeds, Leeds, United Kingdom; ^4^Joint Mass Spectrometry Centre, Chair of Analytical Chemistry, Institute of Chemistry, University of Rostock, Rostock, Germany

**Keywords:** aerobiome, bioaerosols, airborne bacteria, airborne fungi, size-resolved bioaerosols, mineral dust

## Abstract

The atmosphere plays an important role in transporting microorganisms on a global scale, yet the processes affecting the composition of the airborne microbiome, the aerobiome, are not fully outlined. Here we present the community compositions of bacteria and fungi obtained by DNA amplicon-sequencing of aerosol samples collected in a size-resolved manner during nine consecutive days in central Israel. The campaign captured dust events originating from the Sahara and the Arabian deserts, as well as days without dust (“clear days”). We found that the source of the aerosol was the main variable contributing to the composition of both fungal and bacterial communities. Significant differences were also observed between communities representing particles of different sizes. We show evidence for the significant transport of bacteria as cell-aggregates and/or via bacterial attachment to particles during dust events. Our findings further point to the mixing of local and transported bacterial communities, observed mostly in particles smaller than 0.6 μm in diameter, representing bacterial single cells. Fungal communities showed the highest dependence on the source of the aerosols, along with significant daily variability, and without significant mixing between sources, possibly due to their larger aerodynamic size and shorter atmospheric residence times. These results, obtained under highly varied atmospheric conditions, provide significant assurances to previously raised hypotheses and could set the course for future studies on aerobiome composition.

## Introduction

Desert dust storms have been identified as a significant source for microorganisms in the atmosphere, estimated to emit 700–1,400 Gg of bacteria-carrying particles into the atmosphere per year (Griffin et al., 2006; Griffin, 2007; Burrows et al., 2009a). Dust plumes can travel thousands of kilometers and often display a significantly different microbial community composition than that in their destinations (Peter et al., 2014; Meola et al., 2015; Mazar et al., 2016; Gat et al., 2017; Lang-Yona et al., 2020, 2021). The East Mediterranean basin is prone to dust storms from the surrounding Sahara, Arabian and Syrian deserts. Yet it is also a sink for air-masses from Europe, which are characterized by lower concentrations of particulate matter with an aerodynamic diameter of less than 10 μm (PM_10_) (Ganor et al., 2010). Thus situated, the East Mediterranean provides ample opportunities to distinguish between airborne microbiomes, aerobiomes, representing different aerosol sources, concentrations, trajectories and sizes of airborne particles (Lelieveld et al., 2002).

The composition of the aerobiome can affect human and ecosystem health (Aylor et al., 2011; Lang-Yona et al., 2012, 2014; Rahav et al., 2016) and has, therefore, been the focus of numerous studies in the field of atmospheric sciences (e.g., Tong and Lighthart, 2000; Gonzalez-Martin et al., 2014; Du et al., 2018; Maki et al., 2019; Mhuireach et al., 2019; Tignat-Perrier et al., 2020). The aerobiome is comprised of a dynamic, complex assembly of microorganisms, affected by a myriad of atmospheric, geographic and other parameters. Several previous studies have attempted to identify the parameters affecting the composition of the aerobiome and to assess the degree of their influence on the overall community composition (Mazar et al., 2016; Gat et al., 2017; Tignat-Perrier et al., 2019). Among these parameters, aerosol source and sampled air-mass trajectory were found to be of significance in determining the composition of the aerobiome observed at different sites.

It has been suggested that microorganisms, particularly bacteria, are transported in the atmosphere as single cells, as microbial cell aggregates and/or attached to particles such as desert mineral dust (Shaffer and Lighthart, 1997; Burrows et al., 2009a, b). Fungi, which constitute a significant portion of the aerobiome, are likewise carried as single spores or spore aggregates, yet they differ from bacteria in size and aerodynamic properties (Burrows et al., 2009a; Yamamoto et al., 2012; Prussin et al., 2015). While single bacterial cells can be detected by sampling particles of an aerodynamic diameter circa 1 μm (Burrows et al., 2009a), the aerodynamic diameter of a fungal spore is estimated at around 3 μm (Lacey, 1991; Afanou et al., 2014). Thus, size-resolved aerosol sampling can capture single fungal spores along with aggregated bacteria, and spore fragments together with single bacterial cells. Nonetheless, size-resolved sampling of the aerobiome can assist in identifying transport-associated effects on the composition of the aerobiome, and is also important for health reasons, due to the differential deposition of particles of different size in the respiratory system (Lacey, 1991).

In this study, we conducted an intensive field campaign over 9 consecutive days, during which we collected aerosols in a size-resolved manner. The collected samples represent several air-mass sources and weather conditions, including two dust events. The bacterial and fungal communities of these aerosols were identified using amplicon sequencing, and were comparatively examined. We aimed to compare the aerobiome community compositions representing different air-mass sources, different atmospheric particles’ concentrations, as well as different particle size-classes, in order to better define the hierarchy of these parameters in affecting the aerobiome composition.

## Materials and Methods

### Aerosol Sampling

Aerosol sampling took place at the Weizmann Institute of Science campus in Israel, on the roof of a four-story building (31.9070N, 34.8102E, 80 m above mean sea level). Aerosols were sampled, using sterile polyvinylidene fluoride filters (PVDF, 47 mm, 0.45 μm pore size; Millipore), over nine consecutive days, for 6.8–10.4 h at a time, using a Microorifice Uniform Deposit Impactor (MOUDI, model 143 100-NR; MSP Corporation), with nine size-segregating stages (S1–S9) representing the following median particle diameter (D_50_) values: 10, 5.6, 3.2, 1.8, 1.0, 0.6, 0.3, and 0.18 μm for S1–S9, respectively. All filters collected with the MOUDI were stored in sterile petri slide dishes (Millipore) at −20°C. The MOUDI impactor was operated at a flow rate of 30 L min^–1^; Dates, air-mass sources and PM_10_ concentrations are specified in detail in Table 1.

**TABLE 1:** List of aerosol samples, their sources and PM_10_ concentrations.

Sample name	Date	Duration (h)	Mean PM_10_ (μg⋅m^–3^)	Particles in sample	Air-mass Source
SW	10/25/2018	7.5	200 ± 41	1.25⋅10^9^ (f)	South Western Trajectory, Saharan Desert dust
				8.27⋅10^7^ (i)	
				1.73⋅10^7^ (c)	
NW1	10/26/2018	8.5	15 ± 14	1.38⋅10^8^ (f)	North-Western marine trajectory
				1.04⋅10^7^ (i)	
				1.32⋅10^6^ (c)	
NW2	10/27/2018	8.5	18 ± 14	1.19⋅10^8^ (f)	
				6.15⋅10^6^ (i)	
				7.75⋅10^5^ (c)	
ED1	10/28/2018	7.8	50 ± 11	1.53⋅10^8^ (f)	Eastern, continental trajectory, Syrian and/or Arabian deserts
				1.44⋅10^7^ (i)	
				4.72⋅10^6^ (c)	
ED2	10/29/2018	8.5	69 ± 12	2.78⋅10^8^ (f)	
				4.01⋅10^7^ (i)	
				6.99⋅10^6^ (c)	
ED3	10/30/2018	9.6	88 ± 11	3.93⋅10^8^ (f)	
				6.67⋅10^7^ (i)	
				9.06⋅10^6^ (c)	
ED4	10/31/2018	6.8	65 ± 12	2.49⋅10^8^ (f)	
				3.24⋅10^7^ (i)	
				4.24⋅10^6^ (c)	
E1	11/01/2018	7.7	41 ± 10	2.31⋅10^8^ (f)	
				1.13⋅10^7^ (i)	
				3.18⋅10^6^ (c)	
E2	11/02/2018	10.4	28 ± 9	6.78⋅10^8^ (f)	
				1.25⋅10^7^ (i)	
				3.24y10^6^ (c)	

*After Reicher et al. (n.d.). SW, South West dust event (herein “South West”); NW, clear days with air-mass trajectories from North West (herein “North West”); ED, Dust event with air-mass trajectories from the East (herein “East-Dust”); E, clear days with air-mass trajectories from the East (herein “East”). Particles in sample represents the calculated number of particles of each size-class in each sample, based on OPC measurements (f, Fine; i, Intermediate; c, Coarse).*

### Back Trajectories, PM Loading and Number of Collected Particles

The source of the sampled aerosols and their trajectory to the sampling site were determined as described by Reicher et al. (2019), using the Lagrangian analysis tool LAGRANTO 2.0 (Sprenger and Wernli, 2015), based on wind data from the European Centre for Medium-Range Weather Forecasts, ERA-Interim reanalysis (Dee et al., 2011). Back trajectories are presented in Supplementary Figure 1.

PM_10_ data were taken from a measurement station situated about 1 km from the sampling site. This station is part of the air quality monitoring network of the Israel Ministry of Environmental Protection^1^. Sample data is presented in Table 1. Samples were categorized as dust events if they exceeded the mean daily PM_10_ load of 44 μg⋅m^–3^ (Krasnov et al., 2016).

The particles’ size distribution and concentrations were monitored with a portable optical particle counter (OPC, model 1.109; GRIMM Technologies), which has 31 size channels, ranging between 0.25–32 μm (optical equivalent diameters). The amount of collected particles in each sample was calculated by multiplying the OPC data and the volume of air sampled on each sampling occasion, for each size-class. Total particle amount per size-class is detailed in Table 1.

### DNA Extraction and Sequencing

DNA extraction from the filters was performed using the DNeasy PowerWater kit (QIAGEN) with slight modifications to the manufacturer’s protocol: elution was divided into two steps, each step included adding 50 μL EB solution and then centrifuging at 13,000 *g* for 1 min. The filters were cut in half, with half of each filter kept as a backup at −20°C. Two halves from two sequential MOUDI sampler stages were joined in a single PowerWater^®^ bead-tube.

Particles were classified based on size. MOUDI stages S2 + S3 (D_50_ = 5.6 and D_50_ = 3.2 μm, respectively) represent the *coarse particle size-class*, containing bacterial aggregates, bacteria attached to particles and single fungal cells and spores (Burrows et al., 2009a). Stages S4 + S5 (D_50_ = 1.8 and D50 = 1.0 μm, respectively) refer to the *intermediate particle size-class*, containing single bacterial cells, single fungal spores and cells and fungal fragments (Burrows et al., 2009a). S6 + S7 (D_50_ = 0.6 and D_50_ = 0.3 μm, respectively) refer to the *fine particle size-class*, containing mostly fragments of fungi and bacteria, but likely also intact bacterial cells (Tong and Lighthart, 2000). After extraction, the DNA samples were stored at −20°C for further analysis. Blank filters were extracted and processed alongside samples as a negative control.

For bacterial community composition, amplicon sequencing of the V4 region of the gene encoding the small sub-unit of the ribosome, also referred to as 16S rRNA gene, was conducted using primers 515F (GTGCCAGCMGCCGCGGTAA) and 806R (GGACTACHVGGGTWTCTAAT) (Caporaso et al., 2012). This primer set captures bacteria as well as archaea. However, due to the significantly lower abundance of archaea captured, we refer to this community as bacterial, despite the presence of some archaea in the database. For fungal community composition, amplicon sequencing of the ribosomal Internal Transcribed Spacer (ITS) region was conducted using primers ITS1F (CTTGGTCATTTAGAGGAAGTAA) and ITS2R (GCTGCGTTCTTCATCGATGC) (Bruns and Gardes, 1993). All PCR reactions included a no-template control (ntc) as a negative control. DNA sequencing was conducted at the DNA Sequencing Facility (DNAS), University of Illinois at Chicago (UIC), using Illumina Miseq V3 (300 × 2 cycles). Amplification and sequencing were conducted on blank filter samples, ntcs, and true samples. Raw sequences were uploaded to the NCBI SRA database, project accession number: PRJNA750646^2^.

### Sequence Preprocessing and Statistical Analysis

Sequencing data preprocessing was performed using the DADA2 pipeline (Callahan et al., 2019), version 1.16.0. Primer removal (including primers’ reverse complement sequences) was conducted using cutadapt (Martin, 2011) v.2.4. Taxonomic classification was conducted using Silva (Quast et al., 2013) rRNA v.138 and UNITE (Nilsson et al., 2019; UNITE Community, 2019) databases, for prokaryotes and fungi, respectively. A total of 627,670 and 665,087 sequences of bacteria and fungi, respectively, were then assigned to 5,586 and 1,999 Amplicon Sequence Variants (ASVs), respectively. Raw and processed read counts are presented in Supplementary Table 1. Chloroplast and mitochondria sequences were removed manually from the bacterial dataset. No DNA sequences were detected in ITS sequencing of ntc and blank filter controls. In 16S sequencing, some sequences were detected and assigned to ASVs in ntc and blank filter controls. Analysis of the true samples was repeated with and without these suspected contaminant ASVs, with no significant differences found between the results. As a precaution, the results presented in this study were generated following the exclusion of all suspected ASVs from true samples. A minimum prevalence threshold of two samples and 30 counts per ASV was set for the ITS and 16S sequence analyses. A total of 589 and 2,661 ASVs of ITS and 16S, respectively, passed all filtration steps and were used in the subsequent statistical and community-composition analyses. Prevalence analysis and sequence filtration were conducted using Phyloseq R package, v.1.34.0 (McMurdie and Holmes, 2013).

Statistical and community-diversity analyses were conducted using the R packages Vegan (Oksanen et al., 2007), MaAsLin 2 (Mallick et al., 2020, 2021), Hmisc (Harrell, 2020), tidyverse (Henry et al., 2019), ggpubr (Kassambara, 2020), and ggplot2 (Wickham, 2016). For all statistical analyses, ASV counts were transformed using center log-ratio (clr) transformation, to account for the compositionality of the data (Aitchison, 1982; Gloor et al., 2017), after handling zero counts by way of the Geometric Bayesian multiplicative transformation, using the zCompositions package (Palarea-Albaladejo and Martín-Fernández, 2015). All sample pair-wise distances presented in this study, and used for the distance-based analyses, e.g., Principal Component Analysis (PCA), are Euclidean distances. The analyses of the effect of air-mass source and particle size-class on the composition of the aerobiome was conducted using the MaAsLin2 package, with sampling date defined as a random effect to account for the non-independence of different size-classes of a single sampling event. Clustering analysis was conducted using k-means clustering; the number of clusters was pre-determined using the within-cluster sum of squares method (setting seed to 11092020) using the package factoextra (Kassambara and Mundt, 2020). Alpha diversity indices were calculated, using *mothur* v.1.44.0 (Schloss et al., 2009), on ASV counts by sub-sampling repeatedly. Analysis of molecular variance (AMOVA; Excoffier et al., 1992) was also conducted using *mothur*.

## Results

### PM_10_ Data and Back-Trajectories

Results of back-trajectories modeling for each sampling day are presented in Supplementary Figure 1 and are described by Reicher et al. (n.d.). During the 9 sampling days, air-mass back-trajectories varied significantly, leading to significant changes to atmospheric PM_10_ levels. Two sources were linked with PM_10_ concentrations exceeding 44 μg⋅m^–3^, indicating dust events: the Saharan desert on October 25, and the Arabian desert on October 28–31 (Table 1). These days were also characterized by elevated amounts of collected coarse particles, compared with all other days of the campaign, i.e., a mean of 1.06 × 10^6^ (±6.36 × 10^5^) compared with 2.43 × 10^5^ (±1.27 × 10^5^) coarse particles per hour of sampling, for dust and non-dust events, respectively.

Days of low PM_10_ were characterized by air-mass sources from Northern Europe (October 26th and 27th) and from the Arabian desert (November 1st and 2nd); the latter being often a source for dust storms, did not produce high PM_10_ concentrations on the two last sampling days. Thus, we can examine the effect of high/low PM_10_ conditions on the microbial community composition representing air-masses of a single source.

### Microbial Community Structure

**FIGURE 1:**
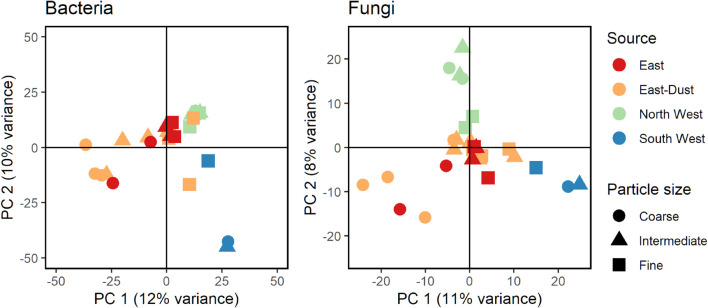
Principal Component Analysis (PCA) analysis of bacterial **(Left)** and fungal **(Right)** communities. Based on Euclidean distances of clr-transformed ASV counts.

Figure 1 presents a PCA of Euclidean distances between samples, based on center log-ratio transformed ASV counts of bacteria and fungi in all aerosol samples. Despite apparent differences in the community structures of bacteria and fungi, some similarities were observed: samples of aerosols originating from the South West (Sahara) were unique, as were aerosols from the North West. The East and East-Dust samples showed similarities in the fungal as well as bacterial community composition (for a list of sources and their definitions, see Table 1). Moreover, in both bacteria and fungi, the fine size-class particles (D_50_ = 0.6 and 0.3 μm) from all sources showed a tendency to cluster more closely together, whereas the coarse size-class-associated communities (D_50_ = 5.6 and 3.2 μm) from all sources were positioned further apart. Yet, while in bacteria, the fine size-class samples formed a cluster close to all the size-classes of the North West samples, in fungi, no such trend was observed.

To assess the relative effect of the different variables, i.e., source, size-class, sampling date, mean PM_10_ and number of collected particles, on the total community composition variance, we performed a PERMANOVA (Anderson, 2001) analysis on the terms: source, size-class, collected particles, mean PM_10_ and sampling date, in this order. Adding sampling date to the list of possibly influential variables allowed us to observe and quantify day-to-day differences in the airborne microbial community, especially since several sampling days experienced air-masses from a single source (e.g., ED_1_, ED_2_, etc.). The permutations were limited within each sampling date to account for the dependence of the different particle size-classes on the sampling event. According to the results, the source of the air-mass was the parameter that most significantly affecting the variability of the bacterial community (*R*^2^ = 0.198, *p*-value = 0.001), followed by sampling date (*R*^2^ = 0.148, *p*-value = 0.001), particle size-class (*R*^2^ = 0.093, *p*-value = 0.001) and PM_10_ concentration (*R*^2^ = 0.042, *p*-value = 0.001). The amount of collected particles did not add significantly to the variance of the bacterial community (*R*^2^ = 0.039, *p*-value = 0.236).

Fungal communities were similarly analyzed and showed similar results. The source of the air-mass was the parameter that most significantly influencing the variability of the fungal community (*R*^2^ = 0.189, *p*-value = 0.001), followed by sampling date (*R*^2^ = 0.167, *p*-value = 0.001), particle size-class (*R*^2^ = 0.096, *p*-value = 0.001) and PM_10_ concentration (*R*^2^ = 0.044, *p*-value = 0.001). The amount of collected particles did not significantly add to the variance of the fungal community (*R*^2^ = 0.030, *p*-value = 0.267). When combined, the selected variables explain only ∼50% of the bacterial and fungal community variances.

A pair-wise comparison between the communities of different sources, regardless of size-class, using AMOVA, revealed that for both bacteria and fungi, all sources displayed significantly different compositions (Benjamini–Hochberg corrected *p*-values < 0.05), except for East and East-Dust (Benjamini–Hochberg corrected *p*-value = 0.4 for bacteria and fungi alike) (Supplementary Table 2).

To better understand the effects of the source of the aerosol on the community composition of each size-class, we divided the samples into the different size-classes and then repeated the comparison between aerosol sources (Supplementary Table 3). The results for bacteria showed that in the coarse and intermediate size-classes, the source of the aerosols significantly affected the bacterial community’s composition (*p*-value = 0.022 and 0.023, respectively), whereas in the fine size-class, no significant difference among the sources was noted (*p*-value = 0.287). In the case of the fungal communities, those associated with coarse and fine size-class particles did not display significant differences between the aerosol sources (*p*-value = 0.2 and 0.4, respectively), while the intermediate size-class did (*p*-value = 0.03).

**FIGURE 2:**
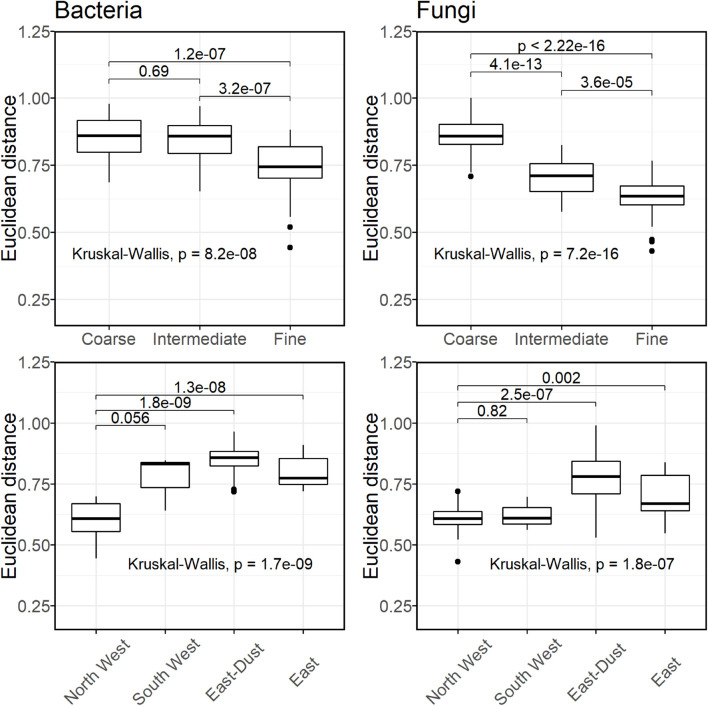
Distribution of Euclidean distances within groups of bacterial **(Left column)** and fungal **(Right column)** communities. Lower Euclidean distances suggest a lower within-group variance.

From the PCA ordination of the different samples as well as the AMOVA results, it was observed that the fine size-class samples from the different sources clustered more closely together than the coarse and intermediate size-classes. To establish this observation, we compared the normalized Euclidean distances of each size-class within itself, and of each source within itself. Thus, only distances between samples of the same size-class or between samples of the same source were considered. For each type of grouping (class-size or source), and for each taxonomic kingdom (bacteria or fungi), we tested the significance of the differences in mean Euclidean distances using the Kruskal–Wallis test. Once we found a significance for a grouping (*p*-value < 0.05), we continued to test pair-wise differences using the Wilcoxon rank-sum test. The results are shown in Figure 2. Bacteria and fungi displayed similar results, namely, the fine size-class-associated communities featured the greatest within-group similarity, observed by significantly lower mean Euclidean distances (0.738 ± 0.101 and 0.629 ± 0.080 for bacteria and fungi, respectively). When grouped according to source, North West and South West displayed the lowest mean Euclidean distances. It is important to note that South West is represented by a single sampling day; thus, statistical significance is not likely.

### Alpha Diversity and Particle Loads

**FIGURE 3:**
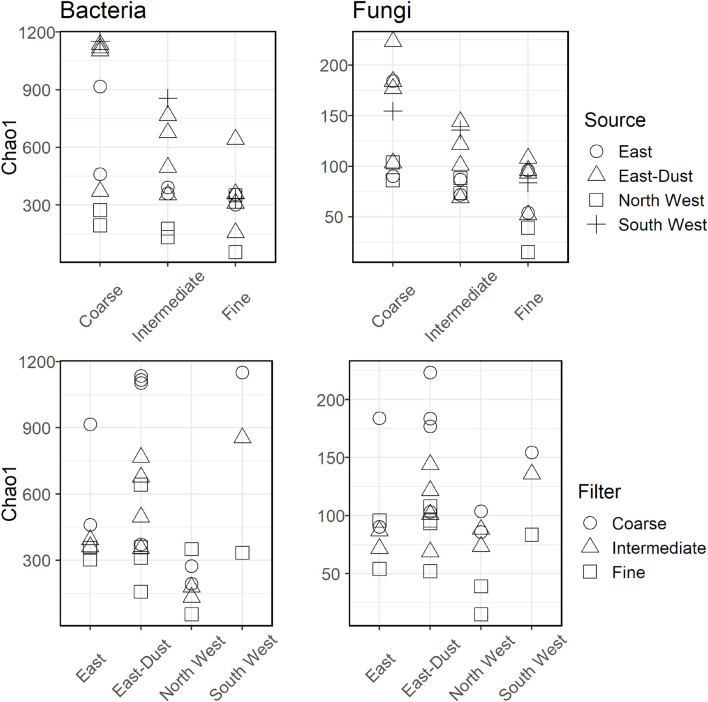
Chao1 diversity for bacteria **(Left)** and fungi **(Right)** over the different aerosol sources and particle size-classes.

Community diversity, expressed by the Chao1 diversity index, was estimated per each sample and is presented in Figure 3. In bacteria and fungi alike, diversity was higher in the coarse size-class particles and in samples that were associated with dust events, i.e., air-masses from South West and East-Dust.

**FIGURE 4:**
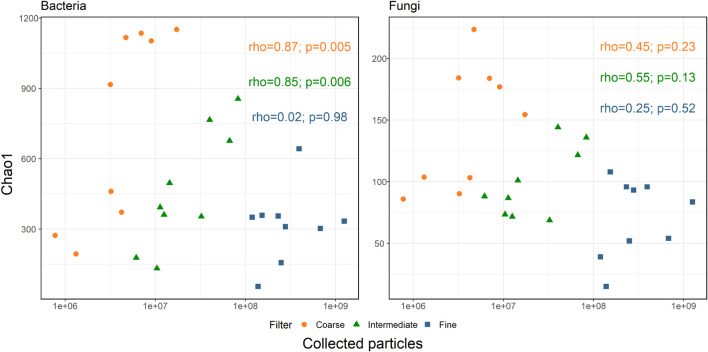
Spearman’s correlation between Chao1 diversity indices and the number of sampled particles per size-class, per sample.

Accordingly, we correlated the Chao1 diversity index to the amount of collected particles of each size-class (Spearman’s correlation, Spearman, 1987). The results are presented in Figure 4. For bacteria, coarse and intermediate size-classes positively correlated with the number of particles (*r* = 0.870, *p*-value = 0.005 and *r* = 0.850, *p*-value = 0.006, respectively). However, no significant correlation with the number of particles was observed for the fine size-class (*r* = 0.020, *p*-value = 0.980). As opposed to bacteria, no correlation was observed between fungal diversity and particle concentration in all size-classes. However, when correlating Chao1 diversity to the number of collected particles over the entire fungal dataset, a significant negative correlation was observed (*r* = −0.38, *p*-value = 0.05). This correlation is likely a biased result of the higher diversity observed in the coarse size-class, which is usually characterized by lower particle concentrations, compared to the low diversity in the fine size-class, which is characterized by significantly higher particle concentrations.

### Effects of Particle Size-Class and Source on the Airborne Microbial Community

We identified PM_10_ loading, number of collected particles, aerosol source and particle size-class as variables that affect or correlate with the composition and diversity of an airborne microbial community. The increasing diversity with particle size in bacterial communities suggested that the intermediate and fine size-classes represented sub-communities of the coarse particles. We examined this hypothesis by way of a NODF nestedness analysis (Almeida-Neto and Ulrich, 2011). Nestedness was examined separately for bacteria and fungi; for all air-mass sources combined; and for each kingdom, separated by air-mass source. The results are presented in Supplementary Table 4. We found that the bacterial communities of the different size-classes were nested as follows: coarse > intermediate > fine (*N* columns = 91.3, *p*-value = 0.005, NODF = 56.7, *p*-value = 0.005) over the entire database. When splitting into the different sources, we found various trends: bacterial communities characterizing North West air-masses were not nested, meaning that the fine size-class was not a subset of the intermediate and coarse size-classes (*N* columns = 37.9, *p*-value = 0.067). All the other sources showed a significant nested structure, with East-Dust and South West samples showing better nestedness than East samples (*N* columns = 81.3, 79.2, and 60.1, *p*-values = 0.023, 0.001, and 0.025, respectively).

In the fungal communities, the total community over all sources displayed a significant nested structure (*N* columns = 82.3, *p*-value = 0.049, NODF = 60.7, *p*-value = 0.039). When divided into the different sources, no single source displayed a significant nested structure of the size-classes (Supplementary Table 4).

### Index Taxa for Different Air-Mass Sources

We also looked for the taxa most affected by the source of the aerosol, while disregarding the particle size-class effect, in both bacteria and fungi, and *vice versa*. This entailed applying a multi-variable linear modeling analysis, in which we treated East samples and East-Dust samples as a single air-mass source, following the PCA and AMOVA observations. The significant ASVs are listed in Supplementary Table 4. Applying a significance threshold of *p*-value = 0.10 (Benjamini–Hochberg correction for multiple comparisons) resulted in 67 bacterial and 44 fungal ASVs that differed between the different air-mass sources, and 26 bacterial and 18 fungal ASVs that significantly differed between the different size-classes.

**FIGURE 5:**
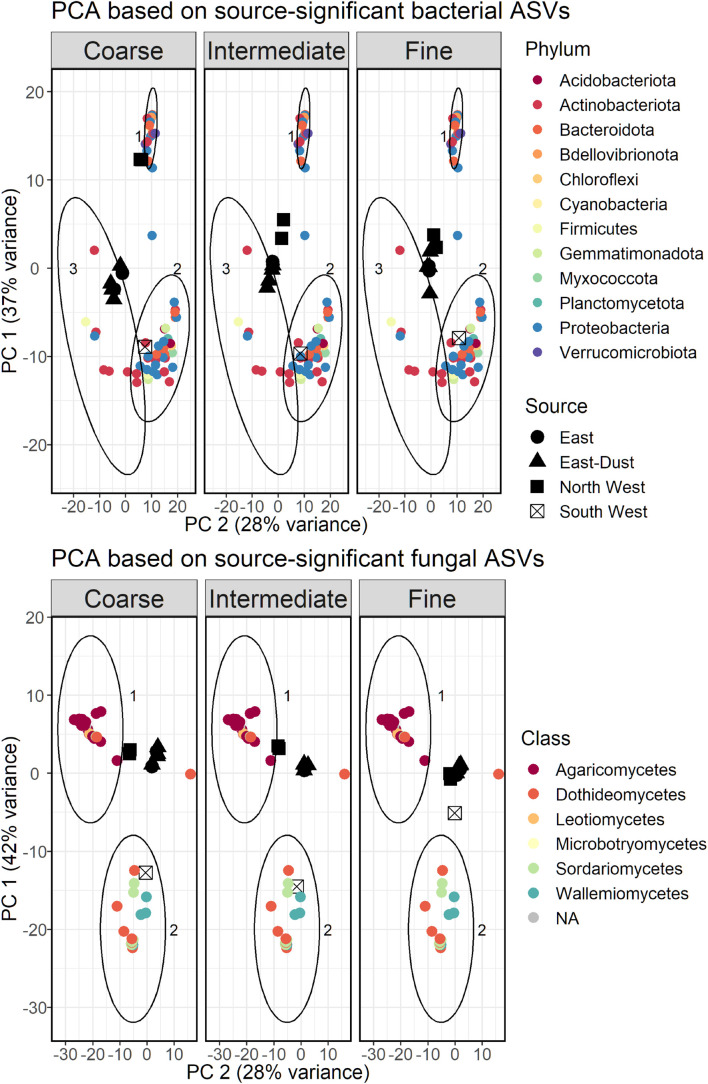
PCA bi-plot based on a subset of bacterial **(Top)** and fungal **(Bottom)** ASVs found to be linked to the source of the air-masses. Clusters were constructed using k-means; clusters 1 and 2 in bacteria and fungi alike represent ASVs that correlated with North West and South West air-masses, respectively; cluster 3 was unique to bacteria and represents ASVs that correlated with East and East-Dust air-masses.

Based only on the source-significant taxa, we repeated the PCA analysis, to better demonstrate which taxa represent each source. The results, as a biplot, are displayed in Figure 5. The bacterial ASVs were divided into three clusters based on *k*-means, with each cluster representing a different air-mass source: cluster 1 consisted of taxa that were more abundant in North West samples, cluster 2 of those more abundant in South West samples, and cluster 3 of those more abundant in East and East-Dust samples. The clusters differed from one another by their taxonomic composition. Cluster 1 (North West) was dominated by Proteobacteria (10 out of 24 ASVs), mostly Gammaproteobacteria, followed by Bacteroidota (5 ASVs). Verrucomicrobiota were found uniquely in this cluster (3 ASVs). Cluster 2 (South West) included a total of 36 ASVs, of which Proteobacteria were most dominant (14 ASVs), mostly Alphaproteobacteria (9 ASVs), followed by Actinobacteriota (9 ASVs) and Bacteroidota (5 ASVs). Gemmatimonadetes were found uniquely in this cluster (2 ASVs). Only 7 ASVs were included in cluster 3, representing East and East-Dust samples, most of which were Actinobacteriota (5 ASVs) and a single ASV each of Alpha-proteobacteria and of Firmicutes.

The same clustering analysis was applied to the fungal ASVs that significantly differed between the various air-mass sources, resulting in five distinct clusters, which were grouped according to the relevant source they represented. Thus, group 1 (consisting of two clusters) represented ASVs that were more abundant in North West samples, and group 2 (consisting of two clusters) represented ASVs that were more abundant in South West samples. As the taxonomic classification of the relevant ASVs showed very low variance at the phylum level, the results are presented at the class level in Figure 5. Group 1 consisted mostly of Agaricomycetes (16 out of 20 ASVs). Group 2, which included a total of 23 ASVs, consisted mostly of Dothideomycetes (10 ASVs), Sordariomycetes (8 ASVs) and Wallemiomycetes (4 ASVs). A single ASV formed a distinct cluster and was not included in either group; it was significantly more abundant in East and East-Dust samples, and was classified as *Mycosphaerella tassiana*, of the class Dothideomycetes.

### Index Taxa for Different Size-Classes

**FIGURE 6:**
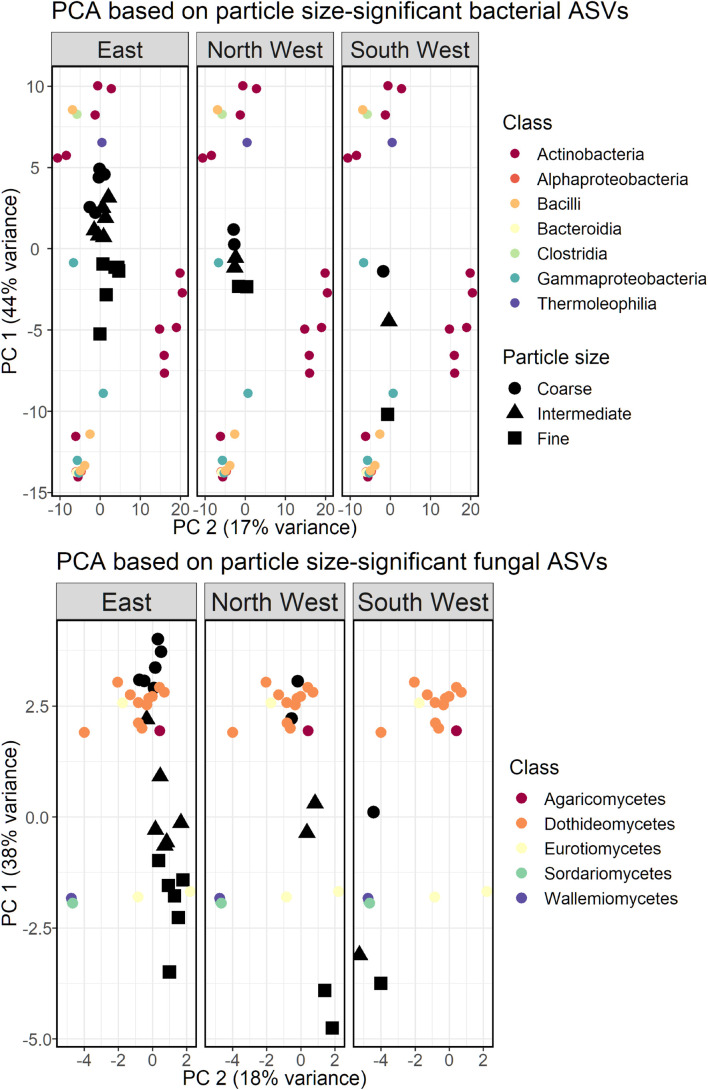
PCA bi-plot based on a subset of bacterial **(Top)** and fungal **(Bottom)** ASVs found to be linked to the size-class of the collected particulate matter. East source refers to East and East-Dust, combined.

We repeated the PCA analysis with bacterial and fungal ASVs that were associated with one or more of the size-classes. Clustering the bacterial and fungal ASVs did not produce coherent results and was therefore omitted. Bacterial classes Gammaproteobacteria, Bacilli and Bacteroidia were associated with the fine size-class, while the coarse size-class was associated with Actinobacteria (class), Thermoleophilia and Clostridia (Figure 6). Although the PCA was based only on size-class-relevant ASVs, North West samples still displayed a smaller variance between the different size-classes, as was observed for the total community analysis.

The PCA biplot of size-class-associated fungal ASVs is presented in Figure 6. Coarse size-class samples of all sources were associated mostly with the fungal class Dothideomycetes (11 out of 13 ASVs), while only 5 ASVs were associated with the fine size-class: two ASVs of Eurotiomycetes, two of Sordariomycetes and a single ASV of Wallemiomycetes.

## Discussion

Particulate matter from various sources, displaying different particulate loadings, was collected in a size-resolved manner over 9 consecutive days. DNA was extracted from these samples, and amplicons of taxonomic marker-genes for bacteria (16S) and fungi (ITS) were sequenced and analyzed. This sampling design enabled a comparative examination of the effect of different parameters, such as source and size-class, on aerobiome composition. This is, to the best of our knowledge, the first study to examine the community composition of both fungi and bacteria in a size-resolved manner with the representation of several air-mass sources.

### Shifts in the Community Structure of Different Air-Masses in Different Size-Classes

We examined the effect of different environmental variables on the aerobiome’s diversity and composition, separating bacteria from fungi, during a 9-days campaign, in which air-masses with substantially different characteristics were sampled. The variables examined were source of the air-mass, particle size-class, mean PM_10_ level and number of collected particles. We also compared the day-to-day differences in community structure between the samples. The sampling design we present is of a relatively small size and is characterized by multiple dependent variables, i.e., it does not include samples of low PM_10_ concentration originating from high dust sources such as the South West, or samples of high PM_10_ concentration from the North West. Therefore, we focused the analysis on two variables that were independent of each other and that showed a relatively high effect on the microbial communities’ variance: the source of the air-mass and the size-class of the collected particles.

Previous studies have associated the source of the air-mass with the aerobiome’s community composition (e.g., Bowers et al., 2011; Peter et al., 2014; Grantham et al., 2015; Gat et al., 2017; Rahav et al., 2019; Tignat-Perrier et al., 2019; Lang-Yona et al., 2020). Fewer studies have compared the aerobiome’s community composition in terms of particles of different size (e.g., Polymenakou et al., 2008; Yamamoto et al., 2012; Helin et al., 2017; Stern et al., 2021). Examining both variables using a single dataset provides a novel opportunity to assess how the microbial community composition of each particle size-class is affected by, or displays, the variance caused by different air-mass sources.

Our results show that microbial communities (bacteria and fungi) associated with fine particles of D_50_ < 0.6 μm were less affected by the source of the air-mass and showed greater within-group similarity than those associated with coarse particles, as observed by the average Euclidean distances between samples of different sources within a single size-class (Figure 2) and by AMOVA tests (Supplementary Table 3). Particles of the fine size-class represent mostly cell fragments (Burrows et al., 2009a), although viable bacterial cells were also previously detected (Tong and Lighthart, 2000). It has been suggested that fragmentation of fungal cells is taxa-specific or growth-stage-specific (Yamamoto et al., 2012; Afanou et al., 2014), resulting in a shift in community composition toward a group of predisposed taxa, regardless of the initial community composition. Moreover, Yamamoto et al. (2012) suggested that Basidiomycota were more abundant in the fine size-class, due to the smaller size of the spores of its three most abundant genera, compared with those of the three most abundant Ascomycota genera. This could explain the tendency of the fine size-class fungal communities to resemble one another, without showing a significant tendency to resemble a specific source.

In bacteria, due to their smaller size, it is possible to find intact cells even in the submicron size-class, but to a lesser extent than in the supermicron size-class (Tong and Lighthart, 2000). As was observed in fungi, the fine size-class bacterial communities displayed greater similarities than did the other two size-classes (Figure 2). Unlike in fungi, the fine size-class communities resembled the bacterial communities representing air-masses from the North West. Moreover, North West communities displayed the greatest within-group similarity, indicating that the different size-classes of this source did not differ much in their community composition. It is interesting to note that the first clear day, following the South West dust event, i.e., October 26, was accompanied by rainfall, which very likely assisted in removing residual particles originating from the South West, thus decreasing the mixing between the two sources. Previous studies conducted at the same location suggested that North West back-trajectories associated with a low PM_10_ concentration represent the local bacterial aerobiome of this area (Mazar et al., 2016; Gat et al., 2017). The results of the bacterial community presented in this study further corroborate this suggestion. No similar indication was observed in this study for fungi.

The air-mass sources of high PM_10_ concentrations (dust events), i.e., South West and East-Dust, representing the Sahara and Arabian deserts, respectively, displayed the highest within-group variability between different size-classes, along with a nested structure, with the fine size-class community being a subset of the intermediate and coarse size-class communities (coarse > intermediate > fine). These observations suggest that bacteria associated with dust events in the Eastern Mediterranean are mostly carried as large aggregates or are attached to dust/soil particles; therefore, they affect the coarse size-class more than the fine size-class bacterial community. During dust transport, the local and transported bacterial communities mix, with different mixing ratios for the different particle size-classes, where the fine size-class is less affected by the dust-borne communities than the coarse size-class, resulting in a relatively stable fine size-class community composition despite the changing sources. This stability in bacterial community composition suggests that this size-fraction is a representative of the local aerobiome. The observed correlation between the bacterial community diversity index and the coarse and intermediate particle amounts also corroborates a mixing effect during dust intrusions. Evidence of mixing between the local aerobiome and transported dust-borne aerobiome was previously shown by Gat et al. (2017), yet it was observed only on the last day of a 3-day long dust storm, and not per each sampling day.

The fungal community sampled in this study exhibited different patterns of community structure from bacteria. No significant nested structure was observed for the different air-mass sources. Moreover, sources associated with high PM_10_ concentrations (South West and East-Dust) did not display a trend of increasing within-group dissimilarity compared with air-mass sources of low PM_10_ concentrations (North West and East). Thus, we suggest that the airborne fungal community is more sensitive to different atmospheric conditions, and its composition responds more quickly than does the bacterial community to changes in the source of the air-mass, resulting in greater shifts between community compositions and less mixing. This is in accordance with the estimated lower residence times of fungal spores in the atmosphere compared with bacteria, i.e., 1 day compared with 3–7.5 days for fungi and bacteria, respectively (Elbert et al., 2007; Burrows et al., 2009a).

### The Effect of Air-Mass Source on Microbial Community Composition

The source of the air-mass exhibited a discernable taxonomic fingerprint, as was reported before by Mazar et al. (2016) and Gat et al. (2017) for bacteria and by Grantham et al. (2015) for fungi. Although only a single sample of the South West trajectory was included in this study, resulting in lower certainty regarding this source, we can compare the results of this sample with previous results of bacterial communities for similar trajectories, reported in Gat et al. (2017). In this study (see Supplementary Table 4), we found that the South West back-trajectory was characterized by the extremophile fungal class Wallemiomycetes (Zalar et al., 2005) and by nitrogen-fixing terrestrial bacteria, such as Rhizobiales (Marín and Arahal, 2014) and Frankiales (Normand, 2006). The order Frankiales, which includes bacteria adapted to harsh environments (Gorbushina, 2007; Sghaier et al., 2016), had a significant presence in similar air-masses sampled at the same location in 2014–2015 (Gat et al., 2017).

Wood-decaying, mushroom-forming fungi from the Agaricomycetes class (Hibbett et al., 2014) were found in North West air-masses, along with human commensal *Enterobacteriaceae* bacteria (Rajilić-Stojanović and de Vos, 2014) and marine-associated bacterial families such as *Halieaceae* (Spring et al., 2013), *Bdellovibrionaceae* and SAR116 clade (Morris et al., 2012). Soil bacteria were also found in the North West air-masses, though to a lesser extent than in the East and South West air-masses.

The East and East-Dust samples were combined for the multivariable association analysis, and were characterized by the abundance of soil-associated bacteria such as *Microvirga* (Marín and Arahal, 2014), *Geodermatophilus* (Normand et al., 2014) and *Blastococcus* (Normand, 2006; Sghaier et al., 2016). The latter also populated similar air-masses sampled in 2015 at the same location (Gat et al., 2017). Human commensals were also found in East and East-Dust air-masses, including *Jeotgalicoccus* (Lory, 2014) and *Corynebacterium* (Spinler, 2015), along with the phyto-pathogenic fungus *M. tassiana* (Petrie and Vanterpool, 1978).

The unique signature of each air-mass provides additional evidence for the significant contribution of the aerosol source to the aerobiome composition in the East Mediterranean. Moreover, as some of these microorganisms belong to potentially harmful taxonomic groups, better understanding their transport characteristics can provide a better insight into and prediction of pathogens’ transport in the atmosphere, along with the application of strain-specific detection methods (Lang-Yona et al., 2012, 2014; Yamamoto et al., 2012; Elmassry et al., 2020).

### Size-Class Specific Microbial Taxa

Size-resolved aerobiome sampling provided new information on the transport of microorganisms in the atmosphere. We found significant taxonomic differences between the fine and coarse size-classes in both bacteria and fungi (Supplementary Table 4). The bacterial taxa found in the coarse size-class were mostly soil bacteria such as *Geodermatophillus* (Normand, 2006; Gorbushina, 2007), *Brachybacterium* (Stackebrandt et al., 2014), *Pseudonocardia* (Franco and Labeda, 2014) and *Solirubrobacter* (Albuquerque and da Costa, 2014), but also animal and human commensals such as *Lactobacillus* (Turnbaugh et al., 2007) and *Romboutsia* (Gerritsen, 2015) were associated with the coarse size-class.

The fine size-class microbial communities were associated with bacteria such as *Quadrisohaera* (Normand and Benson, 2015), *Rhodocytophaga* (Anandham et al., 2010; Park et al., 2020) and *Planococcaceae* (Shivaji et al., 2014), which are associated with various environments. Some bacteria were also putative bioremediators such as *Pseudolabrys* and *Sphingomonas*. Only five fungal taxa were associated with the fine size-class, including extremophiles *Penicillium brevicompactum* (Pitt, 2006) and *Wallemia hederae* (Jancic et al., 2016).

An important parameter that was not examined in this study is the cell-size of the relevant microorganisms. It is highly likely that the cell size would exert an effect on the community composition of each size-class, yet when attempting to find links between the particles’ size-class and the microorganisms’ cell size, we found high variability between species of a single genus or a single family, in both bacteria and fungi. This prevents us from providing more meaningful observations. We thus leave this analysis for future and more focused studies.

Separating the effects of the source of the air-mass from the size-class resulted in relatively few taxa that were significant in each variant. However, some taxa were found to be of significance to each variable, and it is likely that a larger dataset that also includes larger particles, up to 10 μm in diameter, could provide a detailed picture of the interplay between the source of each air-mass and the microbial communities it represents.

### Co-transport of Microorganisms and Particles

During this sampling campaign, high PM_10_ was linked with dust intrusions, resulting in an increase in the number of sampled particles in all size-classes, but most significantly in the coarse size-class (Reicher et al., n.d.). In this study the bacterial community’s alpha-diversity (expressed as Chao1 index) positively correlated with the particle concentration of the intermediate and coarse size-classes. No correlation was observed between bacterial alpha-diversity and particle concentration in the fine size-class. Since bacterial diversity also increased with the rise in dust intrusions (South West and East-Dust), it can be inferred that bacteria in dust storms tend to travel through the atmosphere attached to other particles or as aggregates.

For fungi, no correlation was found between particle concentration, of all size-classes, and their alpha-diversity. This observation suggests that the different air-mass sources sampled in this study represent similarly diverse fungal communities. Our examination of the fungal alpha-diversity per air-mass source found fungal diversity to be relatively low and constant for air-masses from all sources except East-Dust, where some increase was observed. It is less likely that airborne fungal cells and fungal spores were found as aggregates or attached to mineral particles in this study, merely due to their size, which allows them to be included in the coarse size-class as single cells, unlike the significantly smaller bacteria.

## Conclusion

This study provides further evidence in support of previous suggestions regarding the mode of transport of microorganisms in the atmosphere, and specifically to the parameters affecting the composition of the aerobiome in the East Mediterranean. The results exemplify the differences in atmospheric residence times of bacteria and fungi, and the resulting different mixing observed between microbial communities from local and remote sources. We showed evidence corroborating the transport of bacteria in aggregates and/or attached to particles during dust events, but also the transport of single cells, affecting the community composition sampled in the fine size-class. The greater resemblance of bacterial communities from different sources in the fine size-class to the bacterial communities characterizing North West air-masses was attributed to the mixing of local and transported bacteria. Moreover, we provide further evidence that the bacterial communities found in North Western air-masses form a “local” aerobiome in central Israel. The more rapid and abrupt changes in fungal community composition observed here did not allow us to define such a local fungal community. Future research will combine larger datasets, representing more sampling events, along with visualization methods to better establish the observations made here. Future campaigns should define different size-classes for the collection of fungi and bacteria, due to their different sizes. Deciphering the mechanisms of microbial transport in the atmosphere can impact our understanding of microbial eco-system changes, especially in a world of changing climate.

## Data Availability Statement

Raw sequences were uploaded to the NCBI SRA database, project accession number: PRJNA750646 (https://www.ncbi.nlm.nih.gov/sra/PRJNA750646).

## Author Contributions

DG performed all data analyses and wrote the manuscript. NR designed the campaign and calculated all the back-trajectories, PM_10_ and amount of sampled particles. SS performed the DNA extraction, amplification, and initial quality assessment of the acquired sequences. MA collected the aerosol samples. MT designed the campaign, acquired funds, and participated in aerosol sampling. BW participated in aerosol sampling. RZ acquired funds and assisted in writing the manuscript. YR designed the campaign, acquired funds, and hosted the campaign. All authors contributed to reviewing, editing, and finalizing the manuscript.

## Conflict of Interest

The authors declare that the research was conducted in the absence of any commercial or financial relationships that could be construed as a potential conflict of interest.

## Publisher’s Note

All claims expressed in this article are solely those of the authors and do not necessarily represent those of their affiliated organizations, or those of the publisher, the editors and the reviewers. Any product that may be evaluated in this article, or claim that may be made by its manufacturer, is not guaranteed or endorsed by the publisher.

## Footnotes

1
https://www.svivaaqm.net/

2
https://www.ncbi.nlm.nih.gov/sra/PRJNA750646
