# Supplementary material for: Size-Resolved Community Structure of Bacteria and Fungi Transported by Dust in the Middle East
Source: Front Microbiol. 2021 Nov 10;12:744117. doi: 10.3389/fmicb.2021.744117 (PMC8631519; doi:10.3389/fmicb.2021.744117)
Supplement: Supplementary file 2 [file Table_2.DOCX]

Supplementary Material

# Air-mass back-trajectories for the campaign


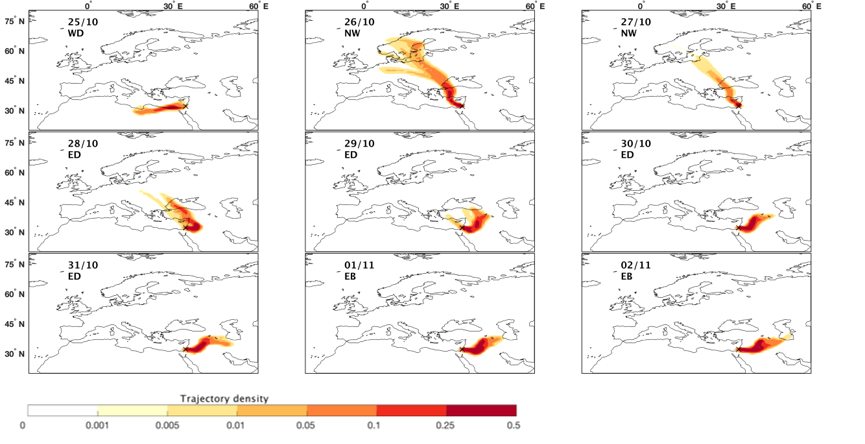


Figure S1: Density and origin of the air-mass trajectories, calculated for the 72 h preceding the time of sampling (After Reicher et al., in prep.).

# Sequence reads summary

Table S1: Number of obtained reads and reads passing DADA2 pipeline for 16S and ITS amplicon sequencing. DADA2 pipeline included the following steps: filtration by read length and quality score, de-noising, merging and chimera detection.

| Sample | 16S reads | 16S reads (passing DADA2 pipeline) | ITS reads | ITS reads (passing DADA2 pipeline) |
| --- | --- | --- | --- | --- |
| SW (fine) | 44245 | 29936 | 37306 | 30304 |
| SW (intermediate) | 59703 | 33670 | 50622 | 40480 |
| SW (coarse) | 64852 | 34462 | 55667 | 40160 |
| NW1 (fine) | 14066 | 11108 | 5907 | 2943 |
| NW1 (intermediate) | 8590 | 5869 | 40603 | 29541 |
| NW1 (coarse) | 18772 | 13709 | 57573 | 41483 |
| NW2 (fine) | 5637 | 4469 | 6269 | 4457 |
| NW2 (intermediate) | 15637 | 12199 | 42922 | 31238 |
| NW2 (coarse) | 19239 | 13944 | 46996 | 33389 |
| ED1 (fine) | 23733 | 17204 | 27313 | 19648 |
| ED1 (intermediate) | 35756 | 26218 | 27784 | 20903 |
| ED1 (coarse) | 59223 | 39000 | 53531 | 38955 |
| ED2 (fine) | 24175 | 17525 | 30182 | 22176 |
| ED2 (intermediate) | 57375 | 41277 | 52074 | 36775 |
| ED2 (coarse) | 61141 | 41824 | 52253 | 38058 |
| ED3 (fine) | 62322 | 40911 | 27503 | 20701 |
| ED3 (intermediate) | 39623 | 28821 | 27566 | 20210 |
| ED3 (coarse) | 60985 | 41416 | 52722 | 38466 |
| ED4 (fine) | 6271 | 4702 | 12910 | 9239 |
| ED4 (intermediate) | 20431 | 14983 | 19857 | 14475 |
| ED4 (coarse) | 19512 | 13931 | 29903 | 21904 |
| E1 (fine) | 18823 | 13287 | 22283 | 16125 |
| E1 (intermediate) | 17914 | 11779 | 15938 | 11698 |
| E1 (coarse) | 61473 | 39525 | 53630 | 38991 |
| E2 (fine) | 20682 | 15463 | 6239 | 4585 |
| E2 (intermediate) | 21973 | 16435 | 18251 | 13599 |
| E2 (coarse) | 56345 | 43644 | 33267 | 24175 |
| Blank filter control | 224 | 173 | 11 | 0 |
| No template control | 321 | 186 | 15 | 0 |

# Taxonomic analysis


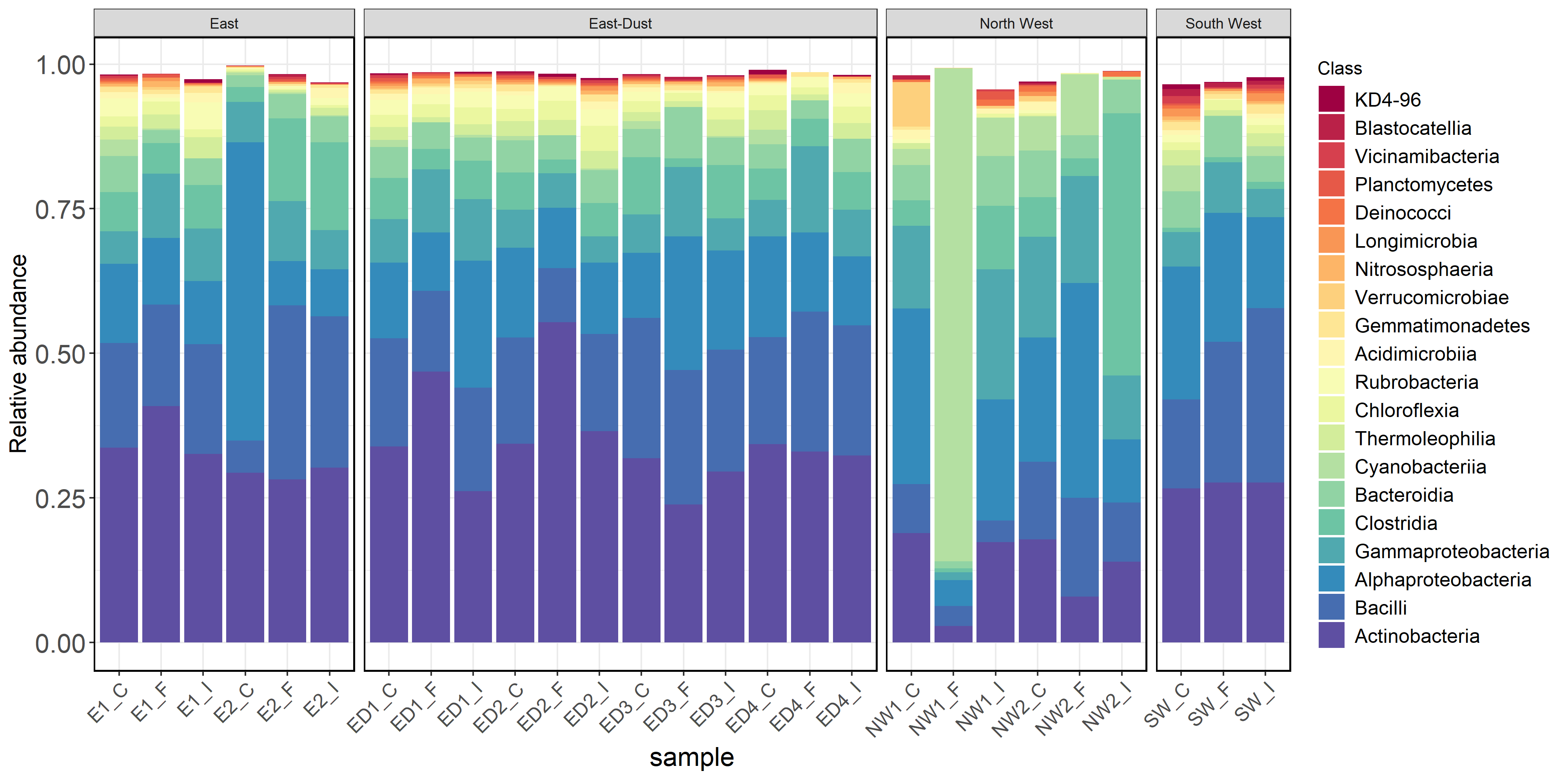


Figure S2: Relative abundance of the 20 most abundant bacterial classes in air samples, separated by air-mass source. The letters “C”, “I” and “F”, on the name of each sample refer to the particles size-class: “Coarse”, “Intermediate” and “Fine”, respectively.


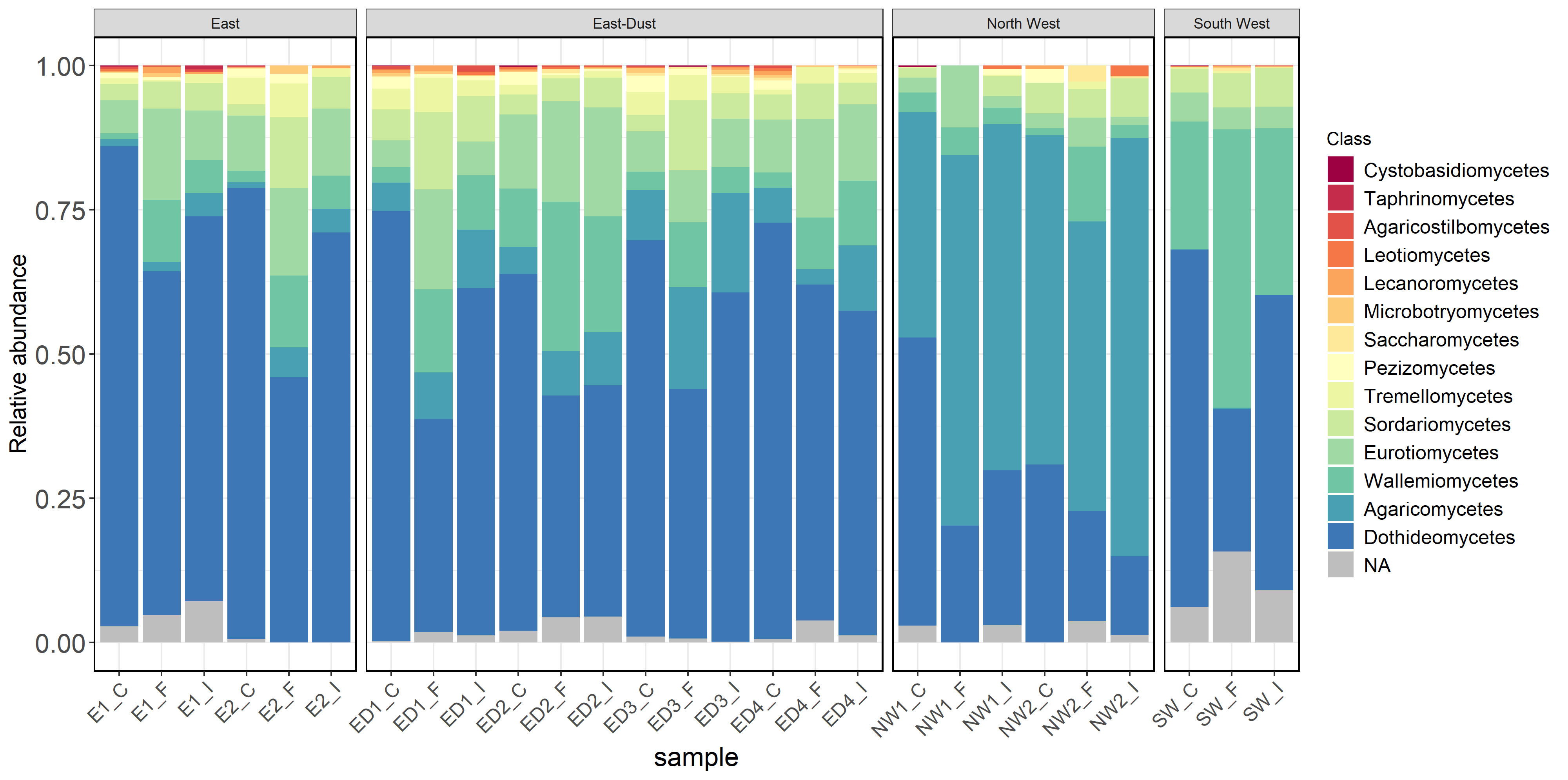


Figure S3: Relative abundance of the fungal classes in air samples, separated by air-mass source. The letters “C”, “I” and “F”, on the name of each sample refer to the particles size-class: “Coarse”, “Intermediate” and “Fine”, respectively.

# Analysis of Molecular Variance (AMOVA) of bacterial and fungal communities

Table S2: AMOVA of bacterial and fungal communities, using samples of all size-classes. Values are Benjamini-Hochberg adjusted p-values.

|  | Bacteria | Fungi |
| --- | --- | --- |
| East-East_Dust | 0.4057 | 0.3626 |
| East-North_West | 0.0028 | 0.0026 |
| East-South_West | 0.0146 | 0.0121 |
| East_Dust-North_West | 0.0028 | 0.0006 |
| East_Dust- South_West | 0.0029 | 0.0021 |
| North_West- South_West | 0.0146 | 0.0121 |

Table S3: AMOVA of bacterial and fungal communities, divided according to the size-class, and comparing the different air-mass sources. Numbers are p-values, describing the likeliness of all the different sources to represent a single microbial community, without referring to pair-wise comparisons between sources.

| Size-class | Bacteria | Fungi |
| --- | --- | --- |
| Fine (D_50_ = 0.3 µm and D_50_ = 0.6 µm) | 0.2872 | 0.3763 |
| Intermediate (D_50_ = 1.0 µm and D_50_ = 1.8 µm) | 0.0227 | 0.0344 |
| Coarse (D_50_ = 3.2 µm and D_50_ = 5.6 µm) | 0.0215 | 0.1541 |

# Nestedness analysis

Nestedness analysis, examining if the different size-classes represent a nested structure according to which, each size-class is a subset of the larger size-class (coarse>intermediate>fine), was conducted using NODF algorithm for bacteria and fungi. An NODF value greater than 50 suggests a nested structure, whereby both species incidence and species composition are nested. An N columns value greater than 50 suggests that species composition shows nestedness between the chosen groups in the defined order. As a null model we randomized the structure of the provided table and iterated this randomization 999 times, enabling the calculation of p-values. This analysis was conducted once for all air-mass sources combined, and once per each air-mass source.

Table S4: Results of nestedness analysis of bacterial and fungal communities, for all air-mass sources combined (Total community), and for each source separately.

|  | Bacteria | | Fungi | |
| --- | --- | --- | --- | --- |
|  | N columns (p-value) | NODF (p-value) | N columns (p-value) | NODF (p-value) |
| Total community | 91.3 (0.005) | 56.7 (0.005) | 82.3 (0.049) | 60.6 (0.039) |
| East | 60.1 (0.025) | 48.1 (0.749) | 54.3 (1.00) | 46.2 (0.109) |
| East-Dust | 81.3 (0.023) | 61.7 (0.023) | 70.6 (0.387) | 57.6 (0.007) |
| South West | 79.3 (0.001) | 57.3 (0.001) | 73.9 (0.173) | 58.2 (0.009) |
| North West | 37.9 (0.067) | 32.0 (0.033) | 67.3 (0.651) | 51.6 (0.509) |
